# Differential expression profiling of tRNA-Derived small RNAs and their potential roles in methamphetamine self-administered rats

**DOI:** 10.3389/fgene.2023.1088498

**Published:** 2023-02-02

**Authors:** Yun Zhou, Qingxiao Hong, Wenjin Xu, Weisheng Chen, Xiaohu Xie, Dingding Zhuang, Miaojun Lai, Dan Fu, Zemin Xu, Majie Wang, Wenhua Zhou, Huifen Liu

**Affiliations:** ^1^ School of Medicine, Ningbo University, Laboratory of Behavioral Neuroscience, Ningbo Kangning Hospital, Ningbo, Zhejiang, China; ^2^ Key Laboratory of Addiction Research of Zhejiang Province, Ningbo, Zhejiang, China

**Keywords:** transfer RNA-derived small RNAs, methamphetamine, addiction, tRF-1-32-Gly-GCC-2-M2, BDNF

## Abstract

Transfer RNA-derived small RNAs (tsRNAs) are a novel class of short, non-coding RNAs that are closely associated with the pathogenesis of various diseases. Accumulating evidence has demonstrated their critical functional roles as regulatory factors in gene expression regulation, protein translation regulation, regulation of various cellular activities, immune mediation, and response to stress. However, the underlying mechanisms by which tRFs & tiRNAs affect methamphetamine-induced pathophysiological processes are largely unknown. In this study, we used a combination of small RNA sequencing, quantitative reverse transcription-polymerase chain reaction (qRT‒PCR), bioinformatics, and luciferase reporter assays to screen the expression profiles and identify the functional roles of tRFs and tiRNAs in the nucleus accumbens (NAc) of methamphetamine self-administration rat models. A total of 461 tRFs & tiRNAs were identified in the NAc of rats after 14 days of methamphetamine self-administration training. Of those, 132 tRFs & tiRNAs were significantly differentially expressed: 59 were significantly upregulated, whereas 73 were significantly downregulated in the rats with methamphetamine self-administration. Decreased expression levels of tiRNA-1-34-Lys-CTT-1 and tRF-1-32-Gly-GCC-2-M2, as well as increased expression levels of tRF-1-16-Ala-TGC-4 in the METH group compared with the saline control were validated by using RT‒PCR. Then, bioinformatic analysis was performed to analyse the possible biological functions of tRFs & tiRNAs in methamphetamine-induced pathogenesis. Furthermore, tRF-1-32-Gly-GCC-2-M2 was identified to target BDNF using the luciferase reporter assay. An altered tsRNA expression pattern was proven, and tRF-1-32-Gly-GCC-2-M2 was shown to be involved in methamphetamine-induced pathophysiologic processes by targeting BDNF. The current study provides new insights for future investigations to explore the mechanisms and therapeutic methods for methamphetamine addiction.

## 1 Introduction

Methamphetamine (METH) is a synthetic amphetamine stimulant of the central nervous system. The United Nations Office on Drugs and Crime (UNODC, 2021) reported that 27 million people used amphetamines worldwide in 2019, suggesting that amphetamine-type stimulant addiction is a growing global public health concern.

METH addiction is a chronic, relapsing brain disorder characterized by compulsive drug-seeking behaviors, persistent cravings, and high rates of relapse. The key brain regions related to mental dependence are dopamine projection from the ventral tegmental area (VTA) to the nucleus accumbens (NAc). The dopamine energy system is currently recognized as the main pathway for drug reward, and the projection from the prefrontal cortex (PFC) to the NAc is a common pathway for stress, drug-related cues or drug-induced reinstatement. Therefore, the NAc plays important roles in drug reinforcement, motivation, and drug seeking.

METH can cause neuronal death, white matter hypertrophy and gliosis, glutamate neurotoxicity, and neuronal dendrite damage in key brain regions (Thompson et al., 2004; Prakash et al., 2017). High-dose METH abuse can also lead to severe neuropsychiatric consequences, including cognitive impairment such as executive function (Hsieh et al., 2014; Kalayasiri et al., 2014), agitation, anxiety, hallucinations, paranoia, and psychosis (Jayanthi et al., 2021). As a cationic lipophilic molecule, METH stimulates the release of newly synthesized catecholamines in the central nervous system and partially prevents their reuptake (Cho and Melega, 2002), resulting in a rewarding effect. Due to structural similarities, METH can also replace and reverse the endogenous functions of dopamine transporter (DAT), norepinephrine transporter (NET), serotonin transporter (SERT) and vesicular monoamine transporter 2 (VMAT-2). METH redistributes the stored monoamines in vesicles into the cytoplasm and leads to the release of dopamine, norepinephrine and serotonin into the synapse, which then stimulate postsynaptic monoamine receptors (Cruickshank and Dyer, 2009). In addition, METH attenuates the metabolism of monoamines by inhibiting monoamine oxidase, which further promotes the accumulation of excess monoamines in synapses (Sulzer et al., 2005). Moreover, METH may induce changes in cholinergic anti-inflammatory pathways, leading to activation of immune-inflammatory pathways and changes in gut microbiota, leading to an increased risk of leaky gut (Prakash et al., 2017). Therefore, METH reward and relapse-inducing factors are complex and are related to genetic, environmental, endocrine factors, etc. The mechanisms of its development (enhancement), maintenance (craving) and relapse cannot be fully explained by independent factors.

Transfer RNA-derived small RNAs (tsRNAs), also known as RNA-derived fragments (tRFs) or tRNA-derived stress-induced RNAs (tiRNAs), are a class of small non-coding RNAs with lengths of 18–40 nucleotides cleaved by transfer RNA (Qin et al., 2020). tRFs & tiRNAs can be divided into i-tRF, tRF-5, tRF-3, tRF-2, and tRF-1; among them, tRF-1 originates from pre-tRNA digested through RNase Z, and the other tRFs originate from mature tRNAs cleaved at various sites (Xie et al., 2020; Tian et al., 2022).

tRFs & tiRNAs function as functional molecules involved in various biological processes and have been shown to mediate global translation through diverse mechanisms; for example, tRFs & tiRNAs regulate the stability of mRNA through conserved complementary sequence matching that is similar to miRNA binding to mRNA (Luo et al., 2018; Jehn et al., 2020). Evidence has demonstrated that tRFs and tiRNAs function by partially or completely binding mRNA with Argonaute 1(Ago1), Ago 3 and Ago 4 to allow mRNA-degrading enzymes to degrade mRNA (Maute et al., 2013; Kumar et al., 2014; Goodarzi et al., 2015; Xie et al., 2020). Moreover, tRFs & tiRNAs act as protein sponges to affect translation by competitively binding to the translation initiation complex to inactive the initiation factor eIF4G/A/E (Gebetsberger et al., 2017; Zhu et al., 2019; Tian et al., 2022). Furthermore, tRFs and tiRNAs have been found to regulate gene expression, epigenetic modifications and rRNA processing (Xie et al., 2020). Finally, tsRNAs guide Ago to mediates translational regulation by exerting structural effects on mRNAs or rRNAs in a Dicer-independent manner (Kuscu et al., 2018; Shi et al., 2019).

In particular, researchers have detected a variety of highly specific and sensitive tRFs & tiRNAs and demonstrated their critical functional roles in the pathophysiological processes of neurological diseases (Qin et al., 2020). For example, two isoforms of tRF5-Pro-AGG (tRF5-Gly-GCC and tRF5-Glu-CTC) all show an enhanced tendency in the postmortem human hippocampus of Alzheimer’s disease patients (Wu et al., 2021). The rsRNA-5S, tsRNA-Tyr and tsRNA-Arg fragments in AD patients are significantly reduced compared with controls (Zhang et al., 2020). Levels of 5′Gly-GCC, 5′Ala-TGC and 5′Glu-CTC are higher in preseizure than in postseizure samples, and these tRNA fragments in plasma can be used to predict seizure risk in patients with epilepsy (Hogg et al., 2019). Patients with Dubowitz syndrome showed the loss of NSUN2-specific methylation at two residues of the aspartate tRNA (Martinez et al., 2012). In addition, the levels of tRNA-Gly and tRNA-Pro were altered by exercise and might be associated with an anxiolytic behavioural phenotype of male offspring and altered levels of small non-coding RNAs in sperm (Short et al., 2017).

Research on alcohol abuse demonstrated that the expression of glycine tRNA-derived fragments (Gly-tRFs) increases in ethanol-fed mice, and inhibition of Gly-tRFs decreases chronic ethanol feeding-induced hepatosteatosis (Zhong et al., 2019). However, tRF & tiRNA biogenesis under METH addiction has been rarely investigated thus far, and it is still unknown whether they are associated with physiological processes and risk factors for METH addiction. Thus, an understanding of the regulatory role of tRFs & tiRNAs will assist in identifying novel biomarkers and potential therapeutic targets for METH addiction.

## 2 Methods

### 2.1 Animals

Adult male Sprague‒Dawley rats (250–300 g) were housed in a temperature- and humidity-controlled room under a reversed 12-h light/dark cycle. The rats had free access to food and water except when specified. All procedures were approved by the Animal Care and Use Committee of the Ningbo Kangning Hospital, and the study was performed strictly in accordance with guidelines for the Care and Use of Laboratory Animals in the National Institutes of Health (NIH) (Publications No. 80-23, revised 1978).

### 2.2 Drugs

METH (>90% purity) was obtained from the National Institute of Forensic Science (Beijing, China) and dissolved in physiological saline. The dosage was 450 mg a day.

### 2.3 Methamphetamine self-administration

Rats underwent right jugular vein cannulation as previously described (Hong et al., 2020). In brief, a silicone catheter (3.5 cm long, 0.5 mm inner diameter, 0.94 mm outer diameter) was inserted into the right external jugular vein. The other end of the catheter (10 cm, pe20) was passed through the dorsal incision and connected to a customized liquid connector fixed on a special jacket. The catheter was irrigated with heparin saline and cefazolin every day to prevent clogging.

After 3 days of recovery from surgery, rats were trained for 4 h daily for 14 consecutive days to self-administer METH under a fixed ratio paradigm in the operant chamber, and the procedure was in accordance with our previous reports (Mei et al., 2020; Xu et al., 2021). In brief, there exist two nose-poke holes in the operant chamber, an active and inactive one. Rats were immediately injected with an infusion of METH (0.5 mg/kg) as a response to the active hole and paired with illumination of the stimulus light as well as the noise of the infusion pump. Then, a 20-s time-out period was followed, during which the number of active lever presses was recorded but METH was not delivered. However, there were no programmed consequences for the inactive nose-poke hole. Stable self-administration required less than 10% variability for the number of active nose-poke responses in rats over the last 3 days.

### 2.4 Tissue collection and RNA extraction

Rats were anaesthetized using Zoletil 50 (1 mL/kg, intraperitoneal) and rapidly decapitated. There were 8 METH self-administered rats and 6 saline controls. Brain tissues were dissected and isolated and then sliced using a rat brain matrix (2.2–0.8 mm according to coordinates from the Paxinos and Watson (1998) rat brain atlas). The NAc was harvested bilaterally using a stainless-steel cannula. Total RNA was collected using TRIzol reagent (Invitrogen, Carlsbad, CA, USA) according to the manufacturer’s instructions. The integrity and quantity of the RNA sample were assessed using a Qubit 4.0 fluorometer (Invitrogen, Life Technologies). High-quality samples were most important for the small RNA sequencing projects. The amount of purified total RNA was approximately 1–2 µg. The RNA integrity (RIN) needed to be greater than 6.5, as detected by a Qubit 4.0 fluorometer (Invitrogen, Life Technologies).

### 2.5 Sequencing library preparation

Some RNA modifications were altered or removed to avoid interfering with small RNA-seq library construction. The following treatments were performed by using the rtStar™ tRFs & tiRNAs Pretreatment Kit (Arraystar, Rockville, MD, USA): 3′-aminoacyl (charged) was deacylated to 3′-OH, 3′-cP (2′, 3′-cyclic phosphate) was removed to yield 3′-OH for 3′adaptor ligation, 5′-OH (hydroxyl group) was phosphorylated to 5′-P for 5′-adaptor ligation, and m1A and m3C were demethylated for efficient reverse transcription.

The cDNA was synthesized and amplified using Illumina’s special reverse transcription and amplification primers according to the manual of the rtStar™ First-Strand cDNA Synthesis Kit (Arraystar, Rockville, MD, USA). The obtained cDNA (approximately 134–160 bp) was purified by SDS‒PAGE.

### 2.6 Library denaturation and dilution

Libraries were absolutely quantified using an Agilent 2100 Bioanalyzer and then denatured and diluted to a concentration of 1.8 pM. Diluted libraries were loaded onto reagent cartridges and forwarded to sequencing run on an Illumina NextSeq 500 system using a NextSeq 500/550 V2 kit (No. FC-404–2005, Illumina) according to the instructions.

### 2.7 Data collection and analysis

Then, sequenced reads were trimmed according to 5′, 3′-adapters, and cutadapt software was used to discard reads with the wrong length (length <14 nt or length >40 nt). The trimmed reads were aligned to the mature tRNA sequences by using NovoAlign software (v2.07.11). The differentially expressed tRFs and tiRNAs were screened based on the count value using the R package edge R. The expression profiling and differential expression analysis were calculated by the average counts per million (CPM), which represents the abundance of tRFs and tiRNAs. Fold change >1.5 and *p*-value <0.05 were considered statistically significant.

### 2.8 Target gene prediction

Target gene prediction of differentially expressed tRFs and tiRNAs integrates miRanda and TargetScan. All seed type sites and sites where m8, 7mer-m8, and 7mer-a1 perfectly match 2–7 nucleotides are considered effective biological targets. TargetScan and miRanda intersections were used to select the final target genes.

### 2.9 Bioinformatics analysis

The biological functions of predicted targets were revealed by Gene Ontology (GO), and genes were mapped to the Kyoto Encyclopedia of Genes and Genome (KEGG) pathways. The *p*-value denotes the significance of the top Gene Ontology terms and pathways correlated to the conditions. In addition, principal component analysis (PCA), correlation analysis and hierarchical clustering were performed. Then, pie plots, Venn plots, scatter plots and volcano plots were drawn.

### 2.10 Verification by qRT‒PCR

Several tRFs & tiRNAs were selected for further verification by qRT‒PCR. These tRFs & tiRNAs were characterized by < 2-fold change values, *p*-value < 0.05, relatively good statistical homogeneity within groups, and higher CMP, and the predicted target genes were associated with addiction or neurological diseases. In total, 4 upregulated (tRF-59-75-Cys-GCA-1-M4, tRF-59-75-Ala-CGC-1-M5, tRF-58-75-Ala-AGC-1, tRF-1-32-Cys-GCA-1-M3) and 3 downregulated (tRF-1-16-Ala-TGC-4, tiRNA-1-34-Lys-CTT-1 and tRF-1-32-Gly-GCC-2-M2) tRFs & tiRNAs were detected under these conditions. The primers designed for amplification of the tRF transcripts are listed in Table 1. U6 was used as a reference, as a small nuclear RNA (snRNA), U6 is more stable than other reference genes for data normalization. In this study, two batches of rat models constructed in different periods were enrolled to verify the sequencing results. There were 18 METH self-administered rats and 16 saline controls, the remaining biological samples of rats that used for RNA-seq were included.

**TABLE 1:** Primers for amplification of the tRF transcripts.

tRFs & tiRNAs	Primer sequence (5′–3′)	Annealing temperature (°C)	Product length (bp)
U6	Forward: 5′GCT​TCG​GCA​GCA​CAT​ATA​CTA​AAA​T 3′	60	89
	Reverse: 5′CGC​TTC​ACG​AAT​TTG​CGT​GTC​AT 3′		
tRF-59-75-Cys-GCA-1-M4	Forward: 5′TCT​ACA​GTC​CGA​CGA​TCT​CCG 3′	60	48
	Reverse: 5′TGC​TCT​TCC​GAT​CTT​GGA​GG 3′		
tRF-59-75-Ala-CGC-1-M5	Forward: 5′GAG​TTC​TAC​AGT​CCG​ACG​ATC​C 3′	60	44
	Reverse: 5′CGA​TCT​TGG​TGG​AGA​TGC​C 3′		
tRF-58-75-Ala-AGC-1	Forward: 5′GAG​TTC​TAC​AGT​CCG​ACG​ATC​T 3′	60	45
	Reverse: 5′CGA​TCT​TGG​TGG​AGA​TGC​T 3′		
tRF-1-32-Cys-GCA-1-M3	Forward: 5′TCC​GAC​GAT​CGG​GGG​TAT​A 3′	60	50
	Reverse: 5′TCC​GAT​CTA​GTC​AAA​TGC​TCT​ACC 3′		
tRF-1-16-Ala-TGC-4	Forward: 5′TCC​GAC​GAT​CGG​GGA​TGT​A 3′	60	43
	Reverse: 5′GTG​TGC​TCT​TCC​GAT​CTA​TTG​A 3′		
tiRNA-1-34-Lys-CTT-1	Forward: 5′CGG​CTA​GCT​CAG​TCG​GTA​GAG 3′	60	45
	Reverse: 5′TGC​TCT​TCC​GAT​CTG​AGT​CTC​AT 3′		
tRF-1-32-Gly-GCC-2-M2	Forward: 5′GAT​CGC​ATG​GGT​GGT​TCA​GT 3′	60	48
	Reverse: 5′CTC​TTC​CGA​TCT​AGG​CGA​GAA​T 3′		

The amplified cDNA was mixed with Arraystar SYBR^®^ Green qPCR Master Mix (ROX+) (AS-MR-006-5, Arraystar). Serially diluted DNA templates were prepared for standard curve drawing. All cDNA samples were configured with a real-time PCR system, including Master Mix, 10 µM PCR-specific primer and cDNA. qRT‒PCR was performed on a Light Cycler^®^ 480 system (Roche Applied Science) in a final volume of 10 µL: 5 µL of Master Mix (Roche Corporation), 1 µL of primer pairs (10 µM), 2 µL of cDNA and 2 µL of DNase/RNase-free water. For establishment of the melting curve after the amplification reaction, the thermocycling conditions were 95°C for 10 s, 60°C for 60 s, and 95°C for 30 s, slowly heating from 60°C to 95°C (the ramp rate was 0.075°C/s).

### 2.11 Luciferase assay

Brain-derived neurotrophic factor (BDNF) was predicted to be one of the target genes of tRF-1-32-Gly-GCC-2-M2, and there are 2 potential target sites in the 3′UTR (Table 2) Then, a dual luciferase experiment was designed to confirm the possible regulatory effect of tRF-1-32-Gly-GCC-2-M2 on BDNF and validate its binding site. The 3′UTR region of BDNF with 1,000 bp length (supplementary data) containing the wild-type (WT) or mutant (MUT) putative binding sites of tRF-1-32-Gly-GCC-2-M2 was constructed and synthesized respectively by HeYuan Biotechnology Co., Ltd. (Shanghai, China), and then cloned into pMIR-report luciferase vectors. Next, pMIR-REPORT Luciferase-BDNF-3′UTR (tRF-1-32-Gly-GCC-2-M2)-WT and pMIR-REPORT Luciferase-BDNF-3′UTR (tRF-1-32-Gly-GCC-2-M2)-MUT1/MUT2 reporters were cotransfected into 293T/17 cells (CL-0469, Procell, China) with tRF-1-32-Gly-GCC-2-M2 mimics or controls (NC) using Lipofectamine 2000. The 293T cell line was obtained from the cell bank of the Chinese Academy of Sciences. Cells were cultured in Dulbecco’s modified Eagle’s medium (DMEM, Gibco, USA) containing 10% foetal bovine serum (FBS, Gibco, USA) in an atmosphere of 5% CO_2_ at 37°C. After 48 h of transfection, luciferase activity was assessed utilizing the Dual-Luciferase Reporter Assay System (Promega, USA).

**TABLE 2:** A schematic of the base pair regions of the BDNF 3′UTR and the predicted binding sites for tRF-1-32-Gly-GCC-2-M2.

Leftmost position of BDNF 3′UTR	Folding energy (in -Kcal/mol)	Sequence type	Heteroduplex	*p*-value
135	−20.2	predicted target sequence 1	GGT​TCT​ACA​ATC​TAT​TTA​TTG​GAC​ATA​TCC​ATG​A	4.96E-02
		target sites	| | | |:: |: | |: | |: |: | | | | |	
		tRF-1-32-Gly-GCC-2-M2	TCC​GCT​CTT​AAG​ATG​G-TGA​CTT​G - GTGGGTACG	
		mutated sequence 1	CCA​AGA​TGT​TAG​ATA​AAT​AAC​CTG​TAT​AGG​TAC​T	
760	−18.5	predicted target sequence 2	CCATGATGTATCT - CC - CTGGTCC - CCCAGGT	2.34E-01
		target sites	: | | | | | | | | | | | | | | | | |	
		tRF-1-32-Gly-GCC-2-M2	TCC​GCT​CTT​AAG​ATG​GTG​ACT​TGG​TGG​GTA​CG	
		mutated sequence 2	GGT​ACT​ACA​TAG​AGG​GAC​CAG​GGG​GTC​CA	

### 2.12 Statistical analysis

SPSS software (version 21.0, Chicago, IL, USA) was used for data analysis, and the results are shown as the mean ± standard error (SEM). GraphPad Prism 8.0 was used for graphing. The 2^−ΔCq^ method was used to analyse the relative expression levels after RT‒PCR assay. Student’s t-test was used to compare significant differences between two groups. *p* < 0.05 was considered statistically significant.

## 3 Results

### 3.1 Altered expression profiles of tRFs & tiRNAs in NAc tissues of methamphetamine self-administered rats

We had uploaded our sequence data to Sequence Read Archive (PRJNA914480, SRA records will be accessible with the following link after the indicated release date 2026-11-07, https://www.ncbi.nlm.nih.gov/bioproject/9414480). The quality score plot of each sample was plotted to examine the sequencing quality. The quality score Q was logarithmically related to the base calling error probability (P): Q = −10 log 10 (P), and a Q score above 30 (>99.9% correct) was considered high-quality data. The proportion of bases (Q ≥ 30) after quality filtering was more than 97%.

**FIGURE 1:**
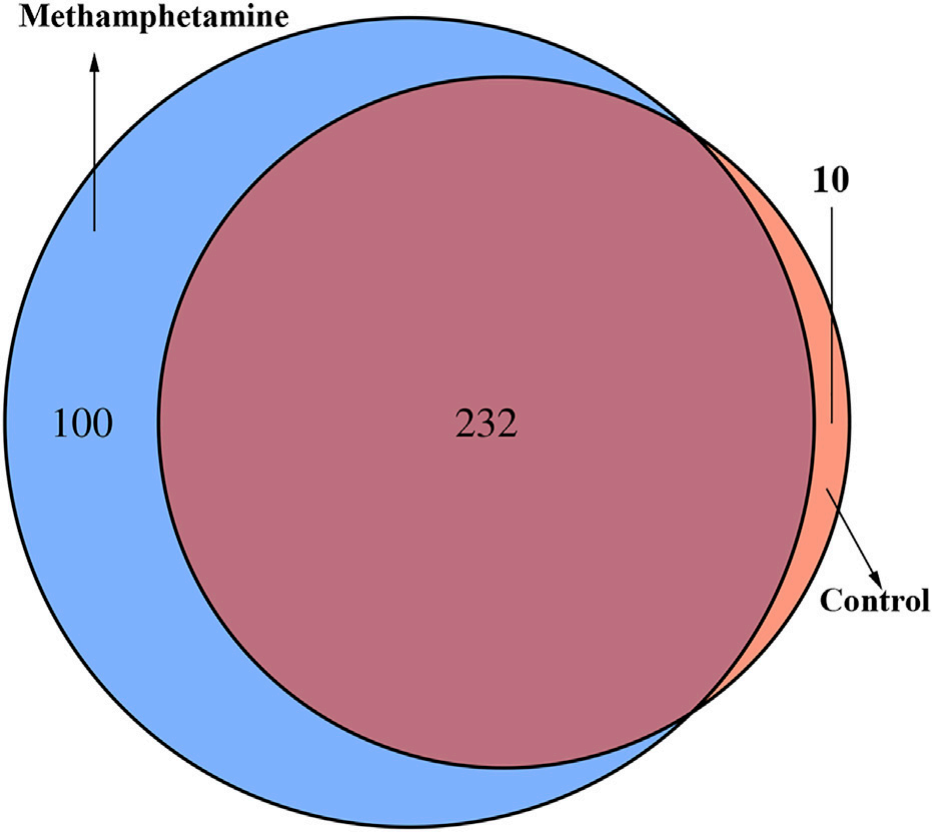
Venn plot to show the total number of identified tsRNAs in the NAc in methamphetamine self-administration and saline control rats.

**FIGURE 2:**
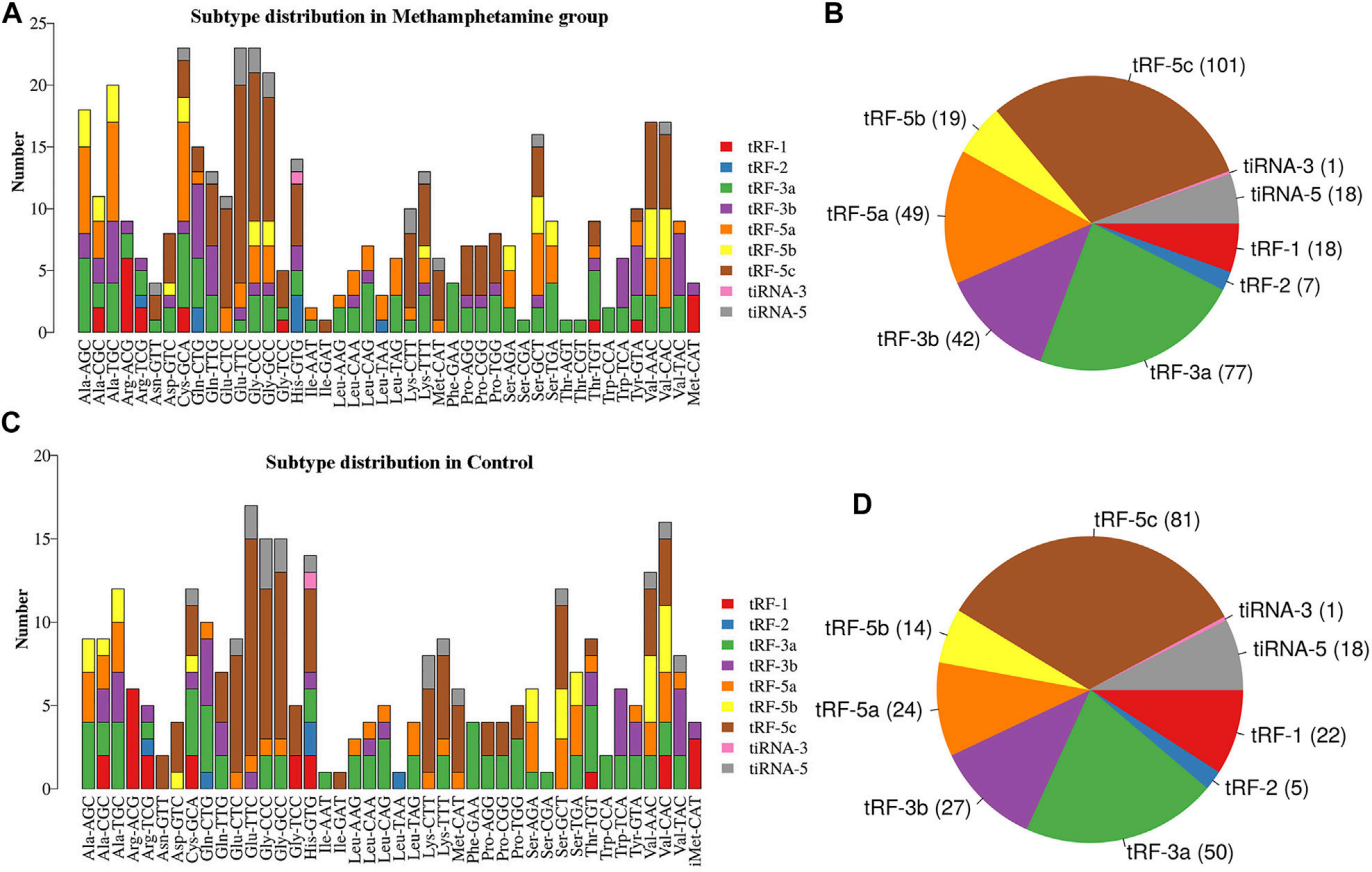
The distribution of tRF and tiRNA subtypes and the number of tsRNA subtypes against tRNA isodecoders. **(A)** and **(B)** Subtype distribution in methamphetamine self-administration rats. **(C)** and **(D)** Subtype distribution in saline control rats. tiRNA: tRNA half, tRF: tRNA-related fragment.

A Venn diagram of the commonly expressed and specifically expressed tRFs & tiRNAs is shown in Figure 1. A total of 232 tRFs & tiRNAs were commonly expressed in both groups, 100 of the tRFs & tiRNAs were specifically expressed in the METH self-administration group, and 10 of the tRFs & tiRNAs were specifically expressed in the control group. Then, the subtypes of tRFs were analysed, as shown in Figures 2A, B, and the number of tsRNA subtypes against tRNA isodecoders was compared *via* stacked bar charts. The anticodon, tRF3-Thr-AGT and tRF3-Thr-CGT only existed in the METH group. The number of tRF-5 and tRF-3 generated from mature tRNAs in the METH self-administration group was significantly higher than that in the control group (*p* < 0.05, Figures 2B–D), while no significant differences were detected in the number of tiRNAs, tRF-1 and tRF-2 between the two groups. tRFs-5 (50.9% and 49%) were the most abundant class of tRFs between the two groups, followed by tRFs-3 (approximately 35.8% and 31.8%).

**FIGURE 3:**
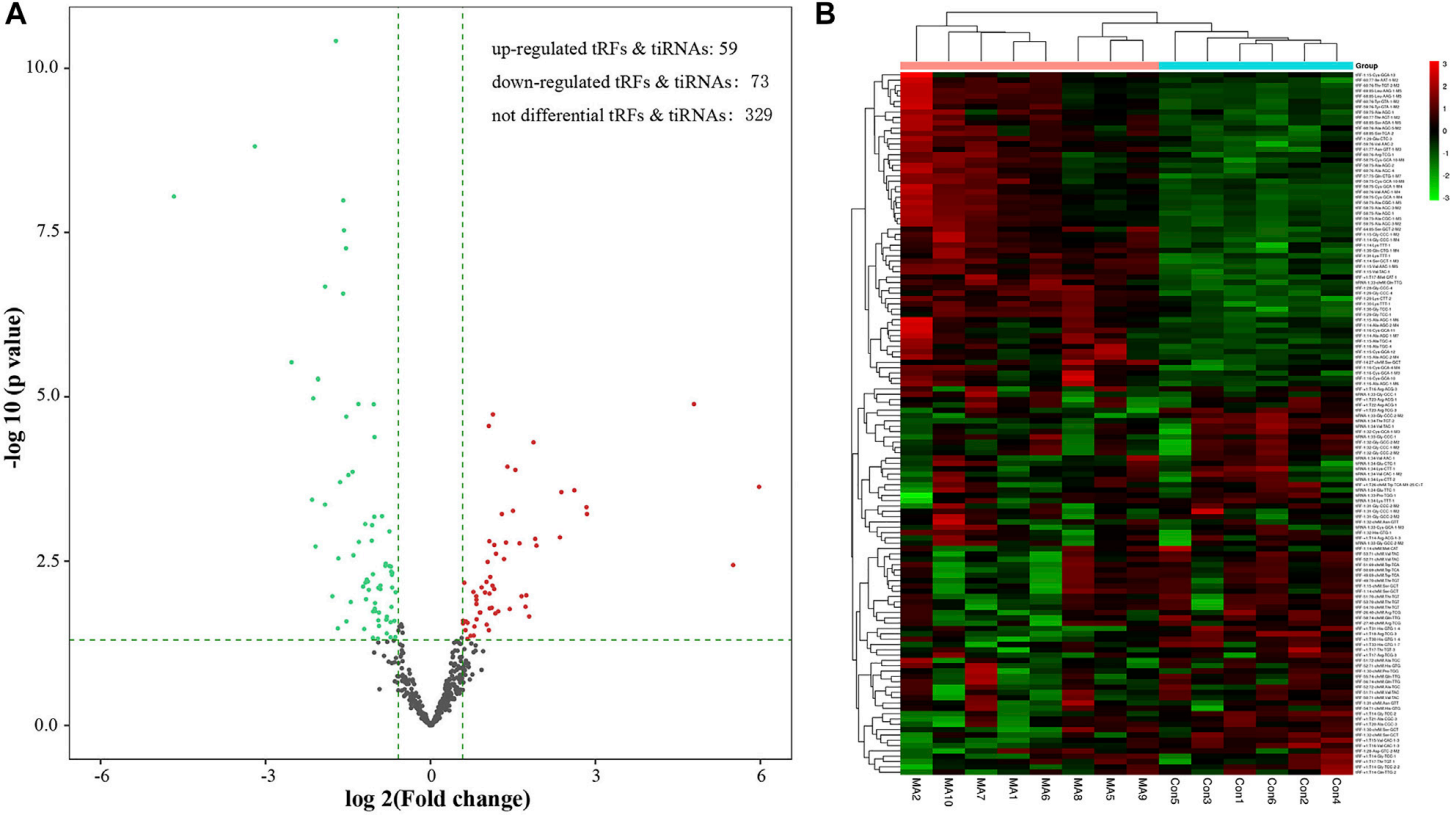
Methamphetamine self-administration alters abundance of tRFs and tiRNAs in the NAc. **(A)** Volcano plot of tRFs and tiRNAs differentially expressed after methamphetamine self-administration, red and green dots indicate upregulated and downregulated tRFs and tiRNAs in methamphetamine self-administration rats, respectively. **(B)** The unsupervised hierarchical clustering heatmap displays relative expression of tRFs and tiRNAs, the color in the panel represents the relative expression level: green represents an expression level below the mean, and red represents an expression level above the mean.

The volcano plots highlight the changes in tRF and tiRNA expression between the two groups. As Figure 3 shows, a total of 132 dysregulated tRFs & tiRNAs were detected between the METH self-administration group and the saline control group. Among them, 59 tRFs & tiRNAs were upregulated, and 73 tRFs and tiRNAs were downregulated. The unsupervised hierarchical clustering heatmap for tRFs & tiRNAs shows that the 132 samples can be arranged into two groups based on the tRF and tiRNA expression levels, which allows us to hypothesize the relationships among samples.

### 3.2 Verification of qRT-PCR

**FIGURE 4:**
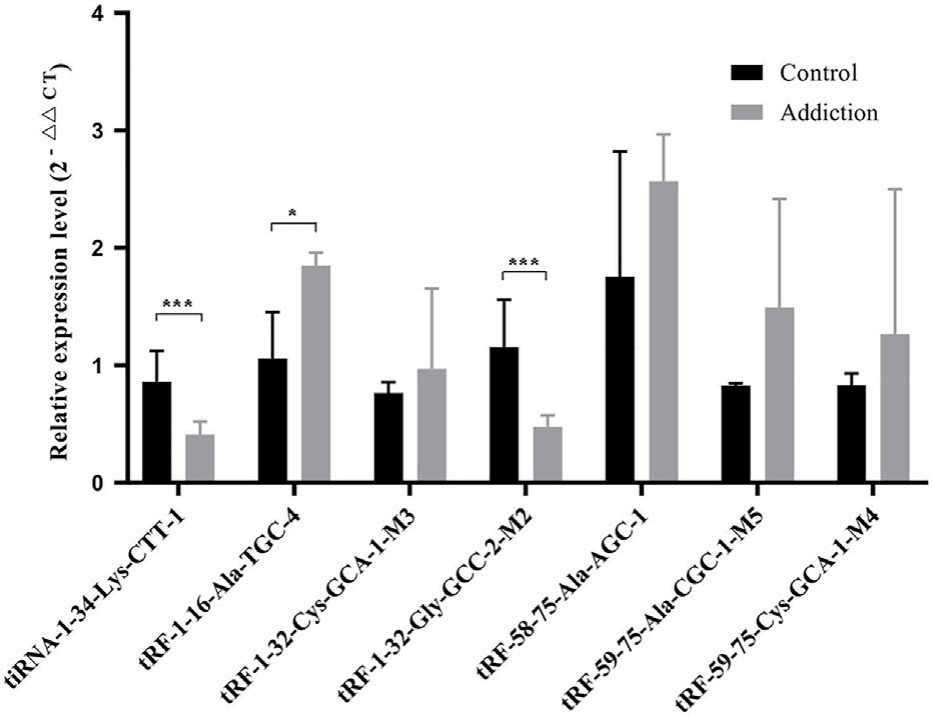
Relative expression levels of seven tRFs and tiRNAs validated by RT-qPCR. Data presents as means ± SEM, *p < 0.05, ***p < 0.0001.

To verify the differentially expressed tRFs & tiRNAs, we screened 7 tRFs & tiRNAs for further verification by qRT‒PCR. The results showed that compared with the controls, the expression levels of tiRNA-1-34-Lys-CTT-1 (*p* = 1.21E-4) and tRF-1-32-Gly-GCC-2-M2 (*p* = 1.49E-5) decreased after METH self-administration (Figure 4), and the results were consistent with the sequencing results. In addition, contrary to the sequencing results, the expression level of tRF-1-16-Ala-TGC-4 increased significantly in the METH self-administration group (*p* = 0.03). No significant difference was found in the other four tRFs between the groups.

### 3.3 Bioinformatic prediction

**FIGURE 5:**
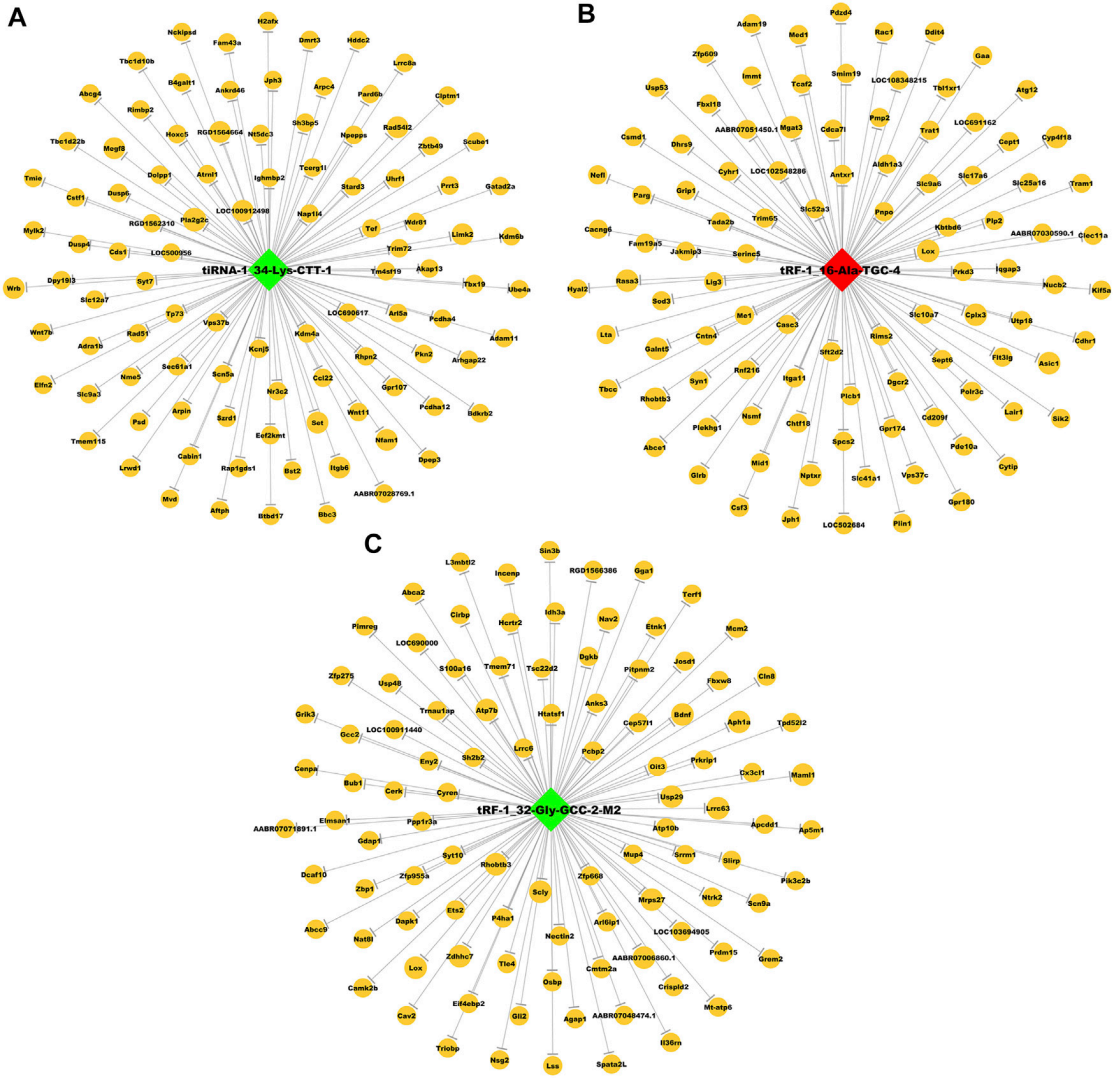
Predicted target mRNAs of three differentially expressed tsRNAs. The tsRNA/mRNA network analysis of **(A)** tiRNA-1-34-Lys-CTT-1, **(B)** tRF-1-16-Ala-TGC-4 and **(C)** tRF-1-32-Gly-GCC-2-M2. Nodes in green and red refer to downregulated and upregulated tsRNAs in sequencing, respectively, nodes in yellow are mRNAs.

To explore the possible roles of tRFs & tiRNAs in addiction, we analysed the target genes of these three differentially expressed tRFs & tiRNAs based on the TargetScan and miRanda algorithms. The numbers of target genes for tiRNA-1-34-Lys-CTT-1, tRF-1-16-Ala-TGC-4 and tRF-1-32-Gly-GCC-2-M2 were 633, 771 and 1,537, respectively. The potential genes had a lower sum of the context + scores used in TargetScan, lower sum of the free energy predicted by miRanda and higher sum of the structure scores used in miRanda. As a result, we constructed tsRNA/mRNA interaction networks using the top 100 target genes of each tsRNA based on the prediction score (Figure 5).

### 3.4 Functional enrichment analysis

**FIGURE 6:**
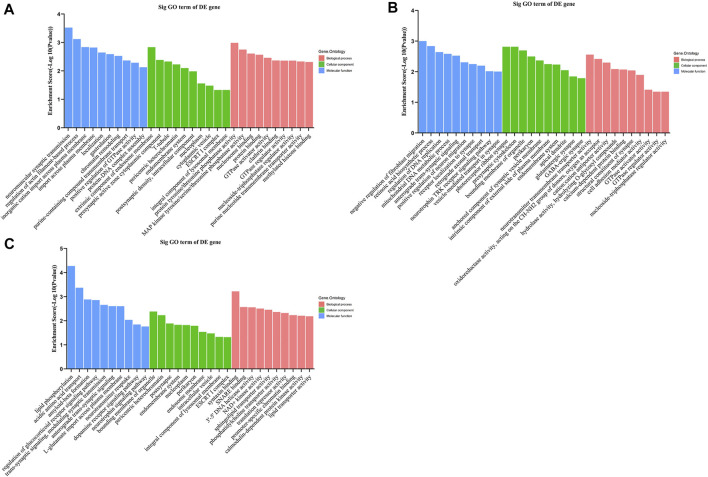
Gene Ontology enrichment analysis shows the 10 related functions changed significantly after methamphetamine self-administration. Gene Ontology enrichment analysis for potential target genes of **(A)** tiRNA-1-34-Lys-CTT-1, **(B)** tRF-1-16-Ala-TGC-4 and **(C)** tRF-1-32-Gly-GCC-2-M2. The horizontal axis shows the biological process (red bars), cellular component (green bars) and molecular function (blue bars) terms, vertical axis shows the enrichment score of each term. GO: Gene Ontology, DE: differently expressed.

Gene Ontology (GO) analysis for target genes of the 3 tRFs & tiRNAs are categorized mostly to biological processes, molecular functions and cellular components. As shown in Figure 6A, the top 2 enriched biological process annotations of tiRNA-1-34-Lys-CTT-1 were neuromuscular synaptic transmission and regulation of actin filament-based processes. The top 2 enriched biological process annotations of tRF-1-16-Ala-TGC-4 were negative regulation of fibroblast migration and retinoic acid biosynthetic process (Figure 6B). The biological process annotations for potential target genes of tRF-1-32-Gly-GCC-2-M2 were related to lipid phosphorylation and acidic amino acid transport, especially, neurotrophin signalling pathway was also enriched (Figure 6C). For the molecular function annotation, according to the GO bioinformatic analysis, GTPase activator activity, GTPase regulator activity and nucleoside-triphosphatase regulator activity were significantly identified for tiRNA-1-34-Lys-CTT-1 and tRF-1-16-Ala-TGC-4, suggesting that elevated expression of these two candidate tRFs is significantly associated with GTPase activity and nucleoside-triphosphatase regulator activity. The top 2 molecular function annotations for potential target genes of tRF-1-32-Gly-GCC-2-M2 were syntaxin binding and SNARE (soluble N-ethylmaleimide-sensitive fusion protein attachment protein receptor) binding. With respect to the cellular component annotation classification, the target genes were mainly associated with the endomembrane system, ESCRT I complex, integral component of lysosomal membrane, bounding membrane of organelle, and pericentric heterochromatin.

**FIGURE 7:**
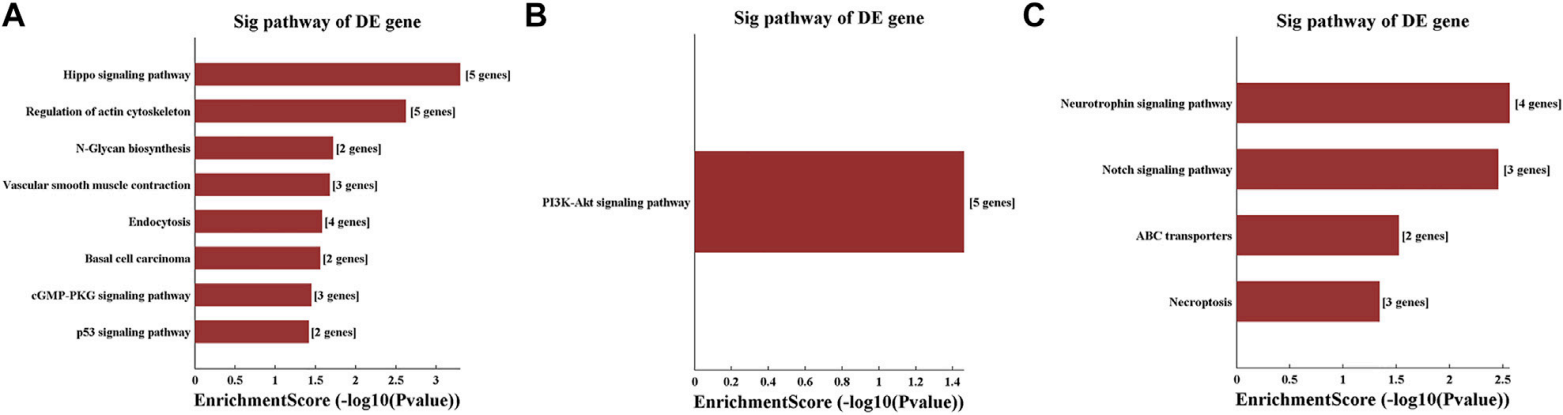
KEGG pathway analysis shows the related pathways changed significantly after methamphetamine self-administration. KEGG pathway analysis for potential target genes of **(A)** tiRNA-1-34-Lys-CTT-1, **(B)** tRF-1-16-Ala-TGC-4 and **(C)** tRF-1-32-Gly-GCC-2-M2. The vertical axis shows the annotated functions of the target genes. The horizontal axis shows the enrichment score (-log10 (*p*-value)) and the gene number of each cluster. KEGG: Kyoto Encyclopedia of Genes and Genome, DE: differently expressed.

The KEGG pathway analysis revealed that the Hippo signalling pathway, regulation of actin cytoskeleton, N-glycan biosynthesis, vascular smooth muscle contraction, endocytosis, basal cell carcinoma, cGMP-PKG signalling pathway and p53 signalling pathway were enriched for tiRNA-1-34-Lys-CTT-1. The PI3K-Akt signalling pathway was enriched for tRF-1-16-Ala-TGC-4. The neurotrophin signalling pathway, Notch signalling pathway, ABC transporters and necroptosis were differentially regulated pathways in the tRF-1-32-Gly-GCC-2-M2 group (Figure 7).

### 3.5 tRF-1-32-Gly-GCC-2-M2 directly targets BDNF

**FIGURE 8:**
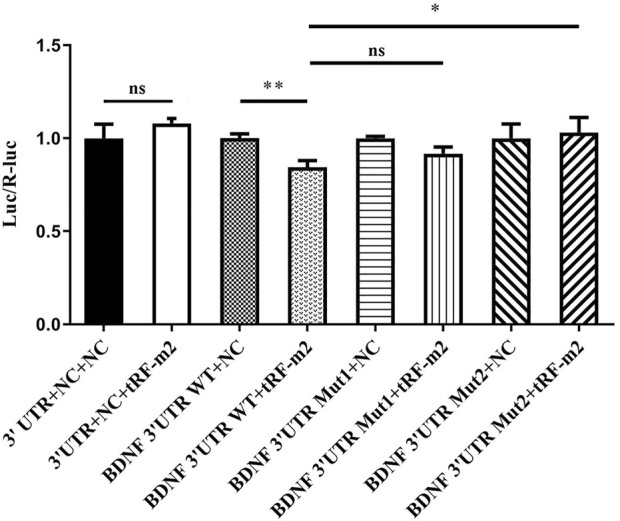
The repressive effect of tRF-1-32-Gly-GCC-2-M2 on the activity of the site of BDNF 3′UTR measured by dual luciferase reporter assays, indicating that tRF-1-32-Gly-GCC-2-M2 directly targeted BDNF. Data presents as means ± SEM, *p < 0.05, ∗∗*p* < 0.001.

Interestingly, for the luciferase reporter assay, a sequence alignment of tRF-1-32-Gly-GCC-2-M2 with the 3′UTR of BDNF is shown. A dual luciferase reporter assay showed the repressive effect of tRF-1-32-Gly-GCC-2-M2 on the activity after binding to the site of BDNF, indicating that tRF-1-32-Gly-GCC-2-M2 directly targets the BDNF 3′UTR (*p* < 0.001, *p* = 0.0035, Figure 8). This regulatory level increased to the initial level of the wild-type control after site 2 mutation (Mut2), indicating that tRF-1-32-Gly-GCC-2-M2 may regulate BDNF expression through binding site 2 (*p* = 0.02). In addition, this regulatory relationship was restored after binding site 1 mutation, with a 7.41% recovery ratio (*p* = 0.069).

## 4 Discussion

In summary, we initially revealed the expression profiles of the tRFs & tiRNAs associated with METH addiction using RNA sequencing. Then, decreased expression levels of tiRNA-1-34-Lys-CTT-1 and tRF-1-32-Gly-GCC-2-M2 and increased expression levels of tRF-1-16-Ala-TGC-4 in the METH group compared with the saline control were validated by using RT‒PCR. In addition, our study indicate that tRF-1-32-Gly-GCC-2-M2 might be involved in the pathophysiological processes of addiction by targeting the BDNF 3′UTR.

In the present study, the expression levels of 132 tRFs and tiRNAs were significantly altered in rat NAc after METH self-administration: 59 upregulated and 73 downregulated tRFs & tiRNAs. The current study offers the first comprehensive assessment of the tRF and tiRNA expression patterns of METH addiction and provides evidence for further investigation. The dysregulated tRFs & tiRNAs here may be beneficially exploited as potential diagnostic biomarkers and/or therapeutic targets to treat METH addiction.

As tRNA-derived small RNAs, tRFs & tiRNAs are not random tRNA degradation products but functional regulatory small molecule fragments with precise sequence structures. tRFs & tiRNAs exhibit a cross-species conserved pattern and circulate in the blood in a stable form, so they are gaining increasing attention for their potential as ideal candidates for neurological diseases (Dhahbi et al., 2013). In recent years, research on tRFs & tiRNAs in the field of drug addiction has attracted considerable attention. For example, 15 tRFs that map to Glu-CTC and Gly-GCC parent tRNAs were altered after chronic intermittent ethanol exposure in mature sperm of mice (Rompala et al., 2018). Interestingly, the metabolic pathways in early embryos were altered following the injection of normal zygotes with sperm tRNA fractions from a high-fat diet (HFD)-fed male mouse model (Chen et al., 2016). Furthermore, sperm tRFs & tiRNAs partially contribute to the enhancement of hedonic behaviors and obesogenic phenotypes in mice induced by a maternal HFD across generations. Microinjection of sperm tRFs & tiRNAs from F1-HFD males into normal zygotes reproduced obesogenic phenotypes and addictive-like behaviors (Sarker et al., 2019).

In addition, many studies have shown the leading role of tRFs & tiRNAs in regulating translational modulation, ribosome biogenesis, stress response, tumorigenesis, stem cell biology, and epigenetic inheritance (Schimmel, 2018; Shi et al., 2019). For example, 155 tRFs & tiRNAs were significantly differentially expressed in the spinal cords of rats 1 day after Spinal Cord Injury (SCI). One candidate tsRNA, tiRNA-Gly-GCC-001, is involved in the MAPK and neurotrophin pathways by targeting BDNF(Qin et al., 2019). Schimmel (Schimmel, 2018) showed that the cleavage of tRNAs at the anticodon by the nuclease angiogenin generates 5′and 3′tsRNAs and that the 5′tsRNAs could inhibit global protein synthesis under stress. Blanco et al. reported that loss of cytosine-5 RNA methylation increases the angiogenin-mediated endonucleolytic cleavage of transfer RNAs (tRNA), resulting in an accumulation of 5’tRNA-derived small RNA fragments. This change leads to decreased protein translation rates, activation of stress pathways, decreased cell size and increased apoptosis in cortical, hippocampal, and striatal neurons (Blanco et al., 2014). The cold shock domain of Y-box-binding protein 1 (YBX1) binds to two tsRNAs (5’tsRNA-Ala and 5’tsRNA-Cys) to form uniquely stable tetramolecular RNA G-quadruplexes (RG4) under stress (such as exposure to reactive oxygen species or UV light). RG4 displaces the translation initiation factor eIF4E/G/A from m7G-capped mRNAs and then disrupts tRNA fragment ability to trigger the formation of stress granules *in vivo* to serve a protective role in human motor neurons (Ivanov et al., 2014; Lyons et al., 2017). NOP2/Sun RNA methyltransferase 2 (NSUN2) can methylate cytosine to 5-methylcytosine (m5C) in various RNAs, including tRNAs(Brzezicha et al., 2006; Auxilien et al., 2012).

In this study, compared with that of the saline control group, the number of tRF-5s and tRF-3s increased significantly in the METH self-administration group. tRF-5s and tRF-3s are derived from functionally conserved mature tRNAs, and tRF-5s are significantly associated with epigenetic inheritance (Zhu et al., 2020). The abundance of tRFs contributes to mRNA decoding in ways that reflect the cell type and its microenvironment. However, how these factors work together to regulate the progression of addiction remains to be understood. In addition, tRF3-Thr-AGT and tRF3-Thr-CGT only existed in the addiction group. This finding suggests that the interaction of target genes with dysregulated tRFs & tiRNAs may selectively increase the available amount of “rare” tRNA isodecoders to a threshold for efficient protein translation. However, the translational changes of the genes affected by these tRNA isodecoder pools are still not clear. Previous studies have shown that tRF3-Thr-AGT targets Btg2, Cd44, Zbp1, etc. Downregulated tRF3-Thr-AGT was shown to be involved in pancreatic acinar intracellular trypsinogen activation (PAITA), which is an important event in the early stage of acute pancreatitis (AP) (Yang et al., 2022). In addition, tRF3-Thr-AGT has been proven to restrain NLRP3-mediated pyroptotic cell death and inflammation in AP models by suppressing ZBP1 expression (Sun et al., 2021). However, there are currently no reports on tRF3-Thr-CGT, and this survey shows that genes affected by this tRNA isodecoder pool may be involved in the pathological process of addiction.

In the present study, qRT‒PCR validation showed that the expression levels of tiRNA-1-34-Lys-CTT-1 (*p* = 1.21E-4) and tRF-1-32-Gly-GCC-2-M2 (*p* = 1.49E-5) decreased after METH self-administration, while the expression level of tRF-1-16-Ala-TGC-4 significantly increased after METH addiction, the target genes of which may be closely related to the occurrence of METH addiction. Through GO and KEGG pathway analyses, the target mRNAs of the differentially expressed tRFs and tiRNAs were found to regulate transsynaptic signalling. A previous study showed that tiRNA-1-34-Lys-CTT-1 and tRF-1-32-Gly-GCC-2-M2 are differentially expressed in vascularized composite allotransplantation model-induced acute rejection (Fang et al., 2022). METH has also been revealed to contribute to dysregulated inflammatory responses that lead to neuroinflammation and subsequent neuronal damage (Chilunda et al., 2022). Therefore, tiRNA-1-34-Lys-CTT-1 and tRF-1-32-Gly-GCC-2-M2 might be associated with synaptic transmission and unstable immune phenotypes. The increased expression of tRF-1-16-Ala-TGC-4 may indicate that the function of related downstream target proteins will be further inhibited, such as the induced disinhibition of negative regulation in fibroblast migration and decrease in retinoic acid biosynthetic process, which might be closely related to the occurrence of METH addiction.

As shown by the GO analysis results, syntaxin binding and SNARE binding are the top two biological processes affected by tRF-1-32-Gly-GCC-2-M2. Syntaxins are nervous system-specific proteins involved in the docking of synaptic vesicles to the presynaptic plasma membrane in several species (Saitsu et al., 2008; Lemos et al., 2010). Tannu et al. revealed that the level of syntaxin-binding protein 3 increases in cocaine-exposed monkeys (Tannu et al., 2010). SNARE protein is essential for the fusion of vacuoles (Veit, 2004). This study suggests a potential use for tRF-1-32-Gly-GCC-2-M2 in the clinic. In the KEGG pathway analysis, the neurotrophin signalling pathway is the top differentially regulated pathway of tRF-1-32-Gly-GCC-2-M2. Therefore, a luciferase reporter assay was performed to detect the relationship between tRF-1-32-Gly-GCC-2-M2 and the critical brain-derived neurotrophic factor BDNF. Interestingly, the results showed that tRF-1-32-Gly-GCC-2-M2 directly targets the 3′UTR of BDNF. A previous study explored whether the serum BDNF concentrations of METH users were significantly higher than those of healthy participants and whether the BDNF concentration was significantly correlated with duration (Moaveni et al., 2022), and a significant correlation between the BDNF Val66Met polymorphism and METH dependence in the overall population was confirmed (He et al., 2020). Our study suggests a potential role of tRF-1-32-Gly-GCC-2-M2 in controlling BDNF expression and explores the therapeutic prospects of tRF-1-32-Gly-GCC-2-M2 for METH addiction by targeting BDNF.

The tRF-1-32-Gly-GCC-2-M2 may be a potential target for the therapeutic treatment of METH addiction. Our future work is to identify the functions of this candidate tRF *in vitro* and *in vivo*.

## Data Availability

The data presented in the study are deposited in the SRA repository (https://www.ncbi.nlm.nih.gov/sra/), accession number PRJNA914480.
